# *N*′-(4-Methoxy­benzo­yl)pyridine-2-carbohydrazide

**DOI:** 10.1107/S1600536810013437

**Published:** 2010-04-24

**Authors:** Hai Zhang, Zuoxiang Wang, Yan Liu

**Affiliations:** a School of Chemistry and Engineering, Southeast University, Nanjing 211189, People’s Republic of China

## Abstract

The crystal structure of the title compound, C_14_H_13_N_3_O_3_, exhibits two inter­molecular N—H⋯O hydrogen bonds.

## Related literature

For general background to the coordination chemistry of pyridine derivatives, see: Koningsbruggen *et al.* (1997 ▶); Klingele & Brooker (2003 ▶); Suksrichavalit *et al.* (2009 ▶). For their biological activity, see: Tozkoparan *et al.* (2000 ▶); Grénman *et al.* (2003 ▶); Alagarsamy *et al.* (2008 ▶); Isloor *et al.* (2009 ▶). For their syntheses, see: Klingsberg (1958 ▶); Potts (1961 ▶).
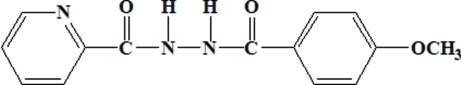


## Experimental

### 

#### Crystal data

C_14_H_13_N_3_O_3_
*M* *_r_* = 271.27
Monoclinic, 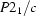

*a* = 14.836 (3) Å
*b* = 11.6078 (17) Å
*c* = 7.6499 (12) Å
β = 97.137 (11)°
*V* = 1307.2 (4) Å^3^
*Z* = 4
Mo *K*α radiation
μ = 0.10 mm^−1^
*T* = 293 K
0.25 × 0.20 × 0.18 mm

#### Data collection

Rigaku SCXmini diffractometer
Absorption correction: multi-scan (*CrystalClear*; Rigaku, 2005 ▶) *T*_min_ = 0.976, *T*_max_ = 0.982
13109 measured reflections
2957 independent reflections
1909 reflections with *I* > 2σ(*I*)
*R*_int_ = 0.053

#### Refinement

*R*[*F* ^2^ > 2σ(*F* ^2^)] = 0.053
*wR*(*F* ^2^) = 0.143
*S* = 1.02
2957 reflections
183 parameters
H-atom parameters constrained
Δρ_max_ = 0.16 e Å^−3^
Δρ_min_ = −0.18 e Å^−3^

### 

Data collection: *CrystalClear* (Rigaku, 2005 ▶); cell refinement: *CrystalClear*; data reduction: *CrystalClear*; program(s) used to solve structure: *SHELXS97* (Sheldrick, 2008 ▶); program(s) used to refine structure: *SHELXL97* (Sheldrick, 2008 ▶); molecular graphics: *SHELXTL* (Sheldrick, 2008 ▶); software used to prepare material for publication: *SHELXL97*.

## Supplementary Material

Crystal structure: contains datablocks I, global. DOI: 10.1107/S1600536810013437/om2329sup1.cif

Structure factors: contains datablocks I. DOI: 10.1107/S1600536810013437/om2329Isup2.hkl

Additional supplementary materials:  crystallographic information; 3D view; checkCIF report

## Figures and Tables

**Table 1:** Hydrogen-bond geometry (Å, °)

*D*—H⋯*A*	*D*—H	H⋯*A*	*D*⋯*A*	*D*—H⋯*A*
N1—H1*A*⋯O1^i^	0.85	2.11	2.9479 (19)	168
N2—H2*A*⋯O2^ii^	0.85	2.13	2.938 (2)	159

Symmetry codes: (i) 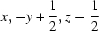; (ii) 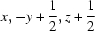.

**Fig. 1.**
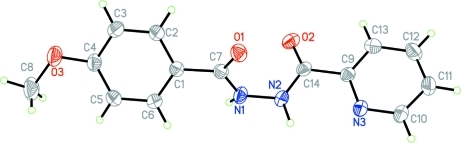
The molecular structure of the title compound with the atomic labelling. Displacement ellipsoids are shown at 30% probability level.

## supplementary crystallographic information

### Comment

As the 1,2,4-triazole ring possesses strong electron donors, the coordination chemistry of 1,2,4-triazole derivatives has gained a great deal of attention in recent years (Koningsbruggen *et al.*, 1997; Klingele & Brooker 2003; Suksrichavalit *et al.*, 2009). Some 1,2,4-triazole compounds have biological activity (Tozkoparan *et al.*, 2000; Grénman *et al.*, 2003; Alagarsamy *et al.*, 2008; Isloor *et al.*, 2009). We report here the crystal structure of the title compound, which can be used to synthesize 3(or 5)-(2-pyridyl)-1,2,4-triazole derivatives (Klingsberg, 1958; Potts, 1961).

The stucture of the title compound is shown in Fig. 1. The structure displays two N—H···O intermolecular hydrogen bonds.

### Experimental

The title compound was prepared by the reaction of 2-picolinyl hydrazide (2.75 g, 20 mmol) with 4-methoxybenzoyl chloride (3.5 g, 20 mmol) in 30 ml *N*,*N*-dimethylacetamide at room temperature. The colorless product was collected by recrystallization from ethanol, and the single crystals suitable for X-ray diffraction were selected.

### Refinement

Positional parameters of all the H atoms were calculated geometrically and were allowed to ride on the C, N atoms to which they are bonded, riding with C—H = 0.93 Å (aromatic), 0.96 Å (methyl) or N—H = 0.85 Å, with Uĩso~(H) = 1.2 or 1.5 times U~eq~(C).

### Crystal data

**Table:**

C_14_H_13_N_3_O_3_	*F*(000) = 568
*M**_r_* = 271.27	*D*_x_ = 1.378 Mg m^−^^3^
Monoclinic, *P*2_1_/*c*	Mo *K*α radiation, λ = 0.71073 Å
*a* = 14.836 (3) Å	Cell parameters from 2772 reflections
*b* = 11.6078 (17) Å	θ = 2.8–27.5°
*c* = 7.6499 (12) Å	µ = 0.10 mm^−^^1^
β = 97.137 (11)°	*T* = 293 K
*V* = 1307.2 (4) Å^3^	Block, colorless
*Z* = 4	0.25 × 0.20 × 0.18 mm

### Data collection

**Table:**

Rigaku SCXmini diffractometer	2957 independent reflections
Radiation source: fine-focus sealed tube	1909 reflections with *I* > 2σ(*I*)
graphite	*R*_int_ = 0.053
CCD_Profile_fitting scans	θ_max_ = 27.5°, θ_min_ = 2.8°
Absorption correction: multi-scan (*CrystalClear*; Rigaku, 2005)	*h* = −19→19
*T*_min_ = 0.976, *T*_max_ = 0.982	*k* = −15→14
13109 measured reflections	*l* = −9→9

### Refinement

**Table:**

Refinement on *F*^2^	Secondary atom site location: difference Fourier map
Least-squares matrix: full	Hydrogen site location: inferred from neighbouring sites
*R*[*F*^2^ > 2σ(*F*^2^)] = 0.053	H-atom parameters constrained
*wR*(*F*^2^) = 0.143	*w* = 1/[σ^2^(*F*_o_^2^) + (0.0677*P*)^2^] where *P* = (*F*_o_^2^ + 2*F*_c_^2^)/3
*S* = 1.02	(Δ/σ)_max_ < 0.001
2957 reflections	Δρ_max_ = 0.16 e Å^−^^3^
183 parameters	Δρ_min_ = −0.18 e Å^−^^3^
0 restraints	Extinction correction: *SHELXL97* (Sheldrick, 2008), Fc^*^=kFc[1+0.001xFc^2^λ^3^/sin(2θ)]^-1/4^
Primary atom site location: structure-invariant direct methods	Extinction coefficient: 0.041 (5)

### Special details

**Table:**

Geometry. All esds (except the esd in the dihedral angle between two l.s. planes) are estimated using the full covariance matrix. The cell esds are taken into account individually in the estimation of esds in distances, angles and torsion angles; correlations between esds in cell parameters are only used when they are defined by crystal symmetry. An approximate (isotropic) treatment of cell esds is used for estimating esds involving l.s. planes.
Refinement. Refinement of *F*^2^ against ALL reflections. The weighted *R*-factor wR and goodness of fit *S* are based on *F*^2^, conventional *R*-factors *R* are based on *F*, with *F* set to zero for negative *F*^2^. The threshold expression of *F*^2^ > σ(*F*^2^) is used only for calculating *R*-factors(gt) etc. and is not relevant to the choice of reflections for refinement. *R*-factors based on *F*^2^ are statistically about twice as large as those based on *F*, and *R*- factors based on ALL data will be even larger.

### Fractional atomic coordinates and isotropic or equivalent isotropic displacement parameters (Å^2^)

**Table:**

	*x*	*y*	*z*	*U*_iso_*/*U*_eq_	
C1	0.91128 (11)	0.22439 (14)	0.9042 (2)	0.0411 (4)	
C2	0.97228 (12)	0.13505 (15)	0.8969 (2)	0.0543 (5)	
H2	0.9527	0.0595	0.9075	0.065*	
C3	1.06100 (13)	0.15577 (17)	0.8744 (3)	0.0598 (5)	
H3	1.1007	0.0944	0.8668	0.072*	
C4	1.09167 (12)	0.26761 (16)	0.8629 (3)	0.0526 (5)	
C5	1.03233 (13)	0.35724 (16)	0.8720 (3)	0.0569 (5)	
H5	1.0525	0.4328	0.8656	0.068*	
C6	0.94239 (12)	0.33527 (15)	0.8909 (2)	0.0520 (5)	
H6	0.9023	0.3965	0.8946	0.062*	
C7	0.81706 (11)	0.19679 (14)	0.9344 (2)	0.0433 (4)	
C8	1.21797 (14)	0.3917 (2)	0.8418 (4)	0.0946 (9)	
H8A	1.1887	0.4334	0.7421	0.142*	
H8B	1.2819	0.3868	0.8340	0.142*	
H8C	1.2083	0.4311	0.9482	0.142*	
C9	0.52469 (11)	0.13673 (14)	0.8280 (2)	0.0432 (4)	
C10	0.41189 (12)	0.18980 (18)	0.9872 (3)	0.0589 (5)	
H10	0.3898	0.2394	1.0674	0.071*	
C11	0.35667 (13)	0.10217 (18)	0.9177 (3)	0.0635 (6)	
H11	0.2992	0.0919	0.9517	0.076*	
C12	0.38794 (14)	0.03054 (18)	0.7977 (3)	0.0679 (6)	
H12	0.3516	−0.0289	0.7473	0.081*	
C13	0.47394 (13)	0.04696 (16)	0.7517 (3)	0.0572 (5)	
H13	0.4970	−0.0015	0.6712	0.069*	
C14	0.61801 (12)	0.15765 (15)	0.7797 (2)	0.0448 (4)	
N1	0.75270 (10)	0.26729 (12)	0.8514 (2)	0.0500 (4)	
H1A	0.7632	0.3096	0.7655	0.075*	
N2	0.66187 (9)	0.24618 (13)	0.8666 (2)	0.0507 (4)	
H2A	0.6435	0.2881	0.9461	0.076*	
N3	0.49533 (9)	0.20769 (13)	0.9461 (2)	0.0507 (4)	
O1	0.79830 (8)	0.11775 (10)	1.03016 (15)	0.0530 (4)	
O2	0.65007 (9)	0.10076 (11)	0.66877 (17)	0.0590 (4)	
O3	1.18109 (9)	0.27954 (12)	0.8433 (2)	0.0759 (5)	

### Atomic displacement parameters (Å^2^)

**Table:**

	*U*^11^	*U*^22^	*U*^33^	*U*^12^	*U*^13^	*U*^23^
C1	0.0422 (9)	0.0415 (9)	0.0399 (9)	0.0003 (7)	0.0060 (7)	0.0012 (7)
C2	0.0473 (11)	0.0427 (10)	0.0740 (13)	−0.0009 (8)	0.0114 (9)	0.0056 (9)
C3	0.0489 (11)	0.0502 (11)	0.0822 (14)	0.0078 (9)	0.0150 (10)	0.0052 (10)
C4	0.0411 (10)	0.0567 (11)	0.0607 (11)	−0.0028 (8)	0.0090 (8)	0.0078 (9)
C5	0.0511 (11)	0.0447 (10)	0.0752 (14)	−0.0067 (9)	0.0089 (10)	0.0042 (9)
C6	0.0462 (10)	0.0426 (10)	0.0676 (12)	0.0032 (8)	0.0091 (9)	−0.0022 (9)
C7	0.0457 (10)	0.0427 (9)	0.0429 (9)	−0.0025 (8)	0.0117 (8)	−0.0026 (8)
C8	0.0509 (13)	0.0814 (17)	0.153 (3)	−0.0185 (11)	0.0175 (15)	0.0266 (17)
C9	0.0432 (10)	0.0418 (9)	0.0447 (9)	0.0065 (7)	0.0066 (8)	0.0058 (7)
C10	0.0438 (11)	0.0659 (13)	0.0696 (13)	−0.0007 (9)	0.0169 (10)	−0.0102 (10)
C11	0.0418 (10)	0.0664 (13)	0.0831 (14)	−0.0055 (10)	0.0110 (10)	0.0014 (12)
C12	0.0587 (13)	0.0552 (12)	0.0882 (16)	−0.0154 (10)	0.0035 (11)	−0.0051 (11)
C13	0.0620 (12)	0.0468 (10)	0.0631 (12)	0.0005 (9)	0.0095 (10)	−0.0055 (9)
C14	0.0452 (10)	0.0432 (10)	0.0468 (10)	0.0097 (8)	0.0092 (8)	0.0057 (8)
N1	0.0394 (8)	0.0553 (9)	0.0583 (10)	0.0028 (7)	0.0179 (7)	0.0104 (7)
N2	0.0401 (8)	0.0549 (9)	0.0601 (10)	0.0024 (7)	0.0183 (7)	−0.0033 (7)
N3	0.0400 (8)	0.0549 (9)	0.0583 (9)	−0.0009 (7)	0.0105 (7)	−0.0059 (7)
O1	0.0513 (8)	0.0525 (7)	0.0567 (8)	−0.0058 (6)	0.0124 (6)	0.0079 (6)
O2	0.0620 (8)	0.0579 (8)	0.0601 (8)	0.0135 (6)	0.0192 (6)	−0.0035 (6)
O3	0.0428 (7)	0.0719 (10)	0.1157 (13)	−0.0033 (7)	0.0201 (8)	0.0171 (9)

### Geometric parameters (Å, °)

**Table:**

C1—C6	1.375 (2)	C8—H8C	0.9600
C1—C2	1.382 (2)	C9—N3	1.335 (2)
C1—C7	1.480 (2)	C9—C13	1.373 (2)
C2—C3	1.370 (2)	C10—N3	1.331 (2)
C2—H2	0.9300	C10—C11	1.371 (3)
C3—C4	1.382 (3)	C10—H10	0.9300
C3—H3	0.9300	C11—C12	1.362 (3)
C4—O3	1.361 (2)	C11—H11	0.9300
C4—C5	1.370 (3)	C12—C13	1.378 (3)
C5—C6	1.384 (2)	C12—H12	0.9300
C5—H5	0.9300	C13—H13	0.9300
C6—H6	0.9300	C14—O2	1.2179 (19)
C7—O1	1.2276 (19)	C14—N2	1.347 (2)
C7—N1	1.354 (2)	C14—C9	1.496 (2)
C8—O3	1.413 (3)	N1—N2	1.389 (2)
C8—H8A	0.9600	N1—H1A	0.8500
C8—H8B	0.9600	N2—H2A	0.8500
			
C6—C1—C2	118.15 (16)	O2—C14—N2	123.43 (16)
C6—C1—C7	123.11 (15)	O2—C14—C9	122.51 (16)
C2—C1—C7	118.67 (15)	N2—C14—C9	114.04 (14)
C3—C2—C1	121.17 (17)	N3—C9—C13	123.25 (16)
C3—C2—H2	119.4	N3—C9—C14	117.16 (15)
C1—C2—H2	119.4	C13—C9—C14	119.59 (16)
C2—C3—C4	120.13 (17)	N3—C10—C11	123.56 (18)
C2—C3—H3	119.9	N3—C10—H10	118.2
C4—C3—H3	119.9	C11—C10—H10	118.2
O3—C4—C5	124.74 (17)	C12—C11—C10	118.49 (18)
O3—C4—C3	115.84 (17)	C12—C11—H11	120.8
C5—C4—C3	119.42 (17)	C10—C11—H11	120.8
C4—C5—C6	119.96 (17)	C11—C12—C13	119.42 (18)
C4—C5—H5	120.0	C11—C12—H12	120.3
C6—C5—H5	120.0	C13—C12—H12	120.3
C1—C6—C5	121.14 (16)	C9—C13—C12	118.22 (18)
C1—C6—H6	119.4	C9—C13—H13	120.9
C5—C6—H6	119.4	C12—C13—H13	120.9
O1—C7—N1	122.17 (16)	C7—N1—N2	119.30 (14)
O1—C7—C1	122.91 (16)	C7—N1—H1A	121.7
N1—C7—C1	114.89 (15)	N2—N1—H1A	116.1
O3—C8—H8A	109.5	C14—N2—N1	120.53 (14)
O3—C8—H8B	109.5	C14—N2—H2A	127.8
H8A—C8—H8B	109.5	N1—N2—H2A	111.0
O3—C8—H8C	109.5	C10—N3—C9	117.05 (16)
H8A—C8—H8C	109.5	C4—O3—C8	118.60 (16)
H8B—C8—H8C	109.5		

### Hydrogen-bond geometry (Å, °)

**Table:**

*D*—H···*A*	*D*—H	H···*A*	*D*···*A*	*D*—H···*A*
N1—H1A···O1^i^	0.85	2.11	2.9479 (19)	168
N2—H2A···O2^ii^	0.85	2.13	2.938 (2)	159

Symmetry codes: (i) *x*, −*y*+1/2, *z*−1/2; (ii) *x*, −*y*+1/2, *z*+1/2.
